# De novo variants in exomes of congenital heart disease patients identify risk genes and pathways

**DOI:** 10.1186/s13073-019-0709-8

**Published:** 2020-01-15

**Authors:** Cigdem Sevim Bayrak, Peng Zhang, Martin Tristani-Firouzi, Bruce D. Gelb, Yuval Itan

**Affiliations:** 1 Institute for Personalized Medicine, Icahn School of Medicine at Mount Sinai, New York, NY USA; 2 St. Giles Laboratory of Human Genetics of Infectious Diseases, The Rockefeller University, New York, NY USA; 3 Nora Eccles Harrison Cardiovascular Research and Training Institute, University of Utah, Salt Lake City, UT USA; 4 Mindich Child Health and Development Institute, Icahn School of Medicine at Mount Sinai, New York, NY USA; 5 Department of Pediatrics, Icahn School of Medicine at Mount Sinai, New York, NY USA; 6 Department of Genetics and Genomic Sciences, Icahn School of Medicine at Mount Sinai, New York, NY USA

**Keywords:** Congenital heart disease, De novo variants, Trios, Pathway, Enrichment analysis

## Abstract

**Background:**

Congenital heart disease (CHD) affects ~ 1% of live births and is the most common birth defect. Although the genetic contribution to the CHD has been long suspected, it has only been well established recently. De novo variants are estimated to contribute to approximately 8% of sporadic CHD.

**Methods:**

CHD is genetically heterogeneous, making pathway enrichment analysis an effective approach to explore and statistically validate CHD-associated genes. In this study, we performed novel gene and pathway enrichment analyses of high-impact de novo variants in the recently published whole-exome sequencing (WES) data generated from a cohort of CHD 2645 parent-offspring trios to identify new CHD-causing candidate genes and mutations. We performed rigorous variant- and gene-level filtrations to identify potentially damaging variants, followed by enrichment analyses and gene prioritization.

**Results:**

Our analyses revealed 23 novel genes that are likely to cause CHD, including *HSP90AA1*, *ROCK2*, *IQGAP1*, and *CHD4*, and sharing biological functions, pathways, molecular interactions, and properties with known CHD-causing genes.

**Conclusions:**

Ultimately, these findings suggest novel genes that are likely to be contributing to CHD pathogenesis.

## Background

Congenital heart disease (CHD) is the most common type of birth defect affecting ~ 1% of births. There have been increasing efforts to elaborate genetic variation underlying CHD using the advances in high-throughput genomic technologies. De novo variants (DNVs) have been shown to play a major role in severe, early-onset genetic disorders such as neurodevelopmental disorders and CHD, and their contribution in sporadic CHD has been estimated as nearly 8%, increasing to 28% for individuals with CHD plus extra-cardiac anomalies and/or neurodevelopmental delays [1–4]. The genetic causes of sporadic CHD, the most common form of CHD, remain largely unknown [5, 6].

Exome sequencing studies of parent-offspring trios have been successful in providing insights into DNVs and identifying causal genes, therefore extending our understanding of mechanisms underlying human diseases [4, 7]. In recent studies of CHD trios enrolled in the Pediatric Cardiac Genetics Consortium (PCGC) [8], significant enrichment for genes related to histone modification, chromatin modification, transcriptional regulation, neural tube development, and cardiac development and enrichment in pathways including Wnt, Notch, Igf, HDAC, ErbB, and NF-κB signaling have been reported [1–3]. A comprehensive analysis of WES data of a single large CHD cohort (2871 probands including 1204 previously reported trios) was recently performed, where rare inherited recessive and dominant variants were analyzed by comparing observed and expected numbers estimated from the de novo probabilities [9].

**Fig. 1:**
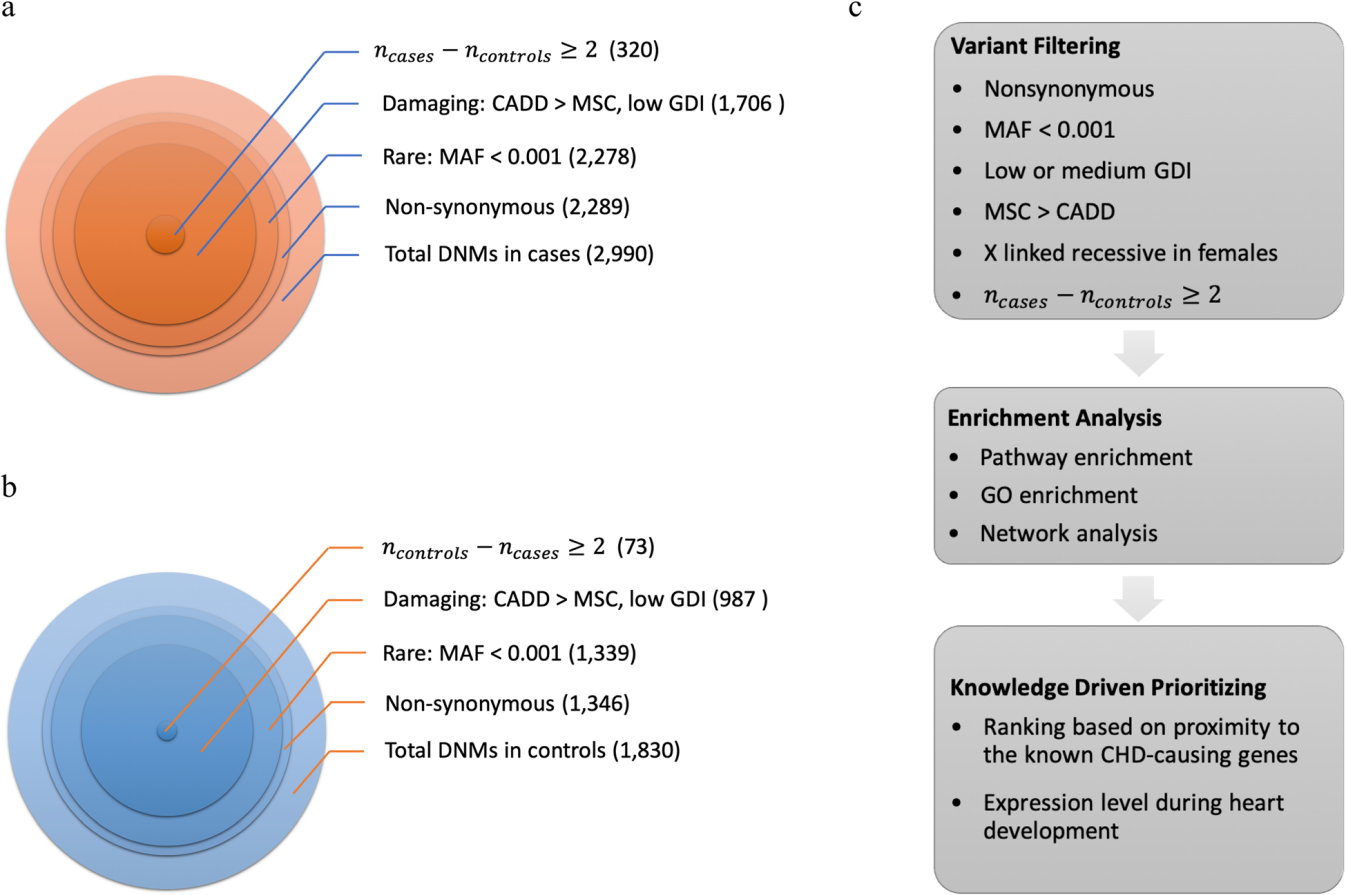
Filtering strategy for de novo variants in **a** 2645 cases and **b** 1789 controls. **c** Identifying likely CHD-causing genes and function-impacting variants

In the present study, we followed a pathway-level approach, which is complementary to the previous approaches of using DNVs to estimate variant rates or to perform gene-level case-control analysis. We performed enrichment analyses on the genes of high-impact DNVs of the same cohort of trios, aiming to identify pathways/networks altered in CHD and novel CHD-causing genes by investigating their shared biological functions, molecular interactions, and properties with known CHD-causing genes. We first rigorously filtered the DNVs in the CHD cohort exomes to determine potentially deleterious ones based on several variant- and gene-level criteria. We then applied enrichment analyses and gene prioritizations based on biological networks, pathways, relatedness to known CHD-causing genes, and heart development tissue expression levels (Fig. 1). We used WES data of 1789 control trios to evaluate the statistical significance of our findings. Assessment of overlapping findings based on several supporting evidence scoring metrics suggested 23 plausible novel genes contributing to CHD.

## Methods

### Patient subjects

De novo variants in patients of CHD and controls were obtained from the recent study of the Pediatric Cardiac Genomics Consortium (PCGC) on a large CHD cohort [9]. We studied 2675 CHD parent-offspring trios recruited to the PCGC and the Pediatric Heart Network (PHN) programs and 1789 control trios comprising parent and unaffected siblings of autism. Each participating subject or their parent/guardian provided informed consent.

PCGC subjects were selected for structural CHD (excluding PDA associated with prematurity, and pulmonic stenosis associated with twin-twin transfusion) and were recruited to the Congenital Heart Disease Genetic Network Study (CHD GENES) [8]. PHN subjects were chosen from the DNA biorepository of the Single Ventricle Reconstruction trial [10]. Controls included 1789 previously analyzed families that include one offspring with autism, one unaffected sibling, and unaffected parents [11]. The permission to access to the genomic data in the Simons Simplex Collection (SSC) on the National Institute of Mental Health Data Repository was obtained. Written informed consent for all participants was provided by the Simons Foundation Autism Research Initiative [12]. Only the unaffected sibling and parents were analyzed in this study. Controls were designated as unaffected by the SSC [11].

Our validation cohort consisted of 559 CHD parent-offspring trios recruited to the PCGC’s CHD GENES whose DNAs had been subjected to WES similar to the discovery case cohort.

The ethnicity and sex distributions of cases and controls are given in Additional file 1: Table S1. Samples with known trisomies or CNVs that are known to be associated with CHD were excluded. Cases include phenotypes with and without extracardiac manifestations or neurodevelopmental deficiency. CHDs were divided into five categories (Additional file 1: Table S2): (i) conotruncal defects (CTD), (ii) d-transposition of the great arteries (d-TGA), (iii) heterotaxy (HTX), (iv) left ventricular outflow tract obstruction (LVO), and (v) other [9].

### Identification of de novo variants

All samples were sequenced at the Yale Center for Genome Analysis following the same protocol as previously described [1]. Genomic DNA from venous blood or saliva was captured using the Nimblegen v.2 exome capture reagent (Roche) or Nimblegen SeqxCap EZ MedExome Target Enrichment Kit (Roche) followed by Illumina DNA sequencing. WES data were processed using two independent analysis pipelines at Yale University School of Medicine and Harvard Medical School (HMS). At each site, sequence reads were independently mapped to the reference genome (hg19) with BWA-MEM (Yale) and Novoalign (HMS) and further processed using the GATK Best Practices workflows [13–15]. Single nucleotide variants and small indels were called with GATK HaplotypeCaller and annotated using ANNOVAR, dbSNP (v138), 1000 Genomes (August 2015), NHLBI Exome Variant Server (EVS), and ExAC (v3) [16, 17]. The MetaSVM algorithm, annotated using dbNSFP (version 2.9), was used to predict deleteriousness of missense variants using software defaults [18, 19]. Variant calls were reconciled between Yale and HMS before downstream statistical analyses.

Relationship between proband and parents was estimated using the pairwise identity-by-descent (IBD) calculation in PLINK [20]. The IBD sharing between the proband and parents in all trios was between 45 and 55%, as expected.

DNVs were called by Yale using the TrioDenovo program [21] and filtered yielding a specificity of 96.3% as previously described [2]. These hard filters include (i) an in-cohort minor allele frequency (MAF) ≤4 × 10^−4^; (ii) a minimum 10 total reads, 5 alternate allele reads, and a minimum 20% alternate allele ratio in the proband if alternate allele reads ≥ 10, or if alternate allele reads is < 10, a minimum 28% alternate ratio; (iii) a minimum depth of 10 reference reads and alternate allele ratio < 3.5% in parents; and (iv) exonic or canonical splice-site variants.

The observed and expected rates for presumably benign synonymous DNVs showed no enrichment in cases or controls [9]. The rate of synonymous DNVs in cases was not different from that in controls.

### The gene sets

The genes in which coding mutations cause isolated or syndromic CHD used in this study are referred to as known CHD-causing genes and include both human and mouse CHD genes. The human CHD gene set was manually curated by members of the Pediatric Cardiac Genomics Consortium [1, 2]. To generate the mouse CHD gene set, mammalian phenotype ontology (MPO) terms potentially relevant to CHD were identified. These were reviewed to remove cardiovascular terms not specific to CHD, such as cardiac dilation/hypertrophy, arrhythmias, and coronary artery disease [22]. Data on the mouse strains associated with these MPO terms (*n* = 1020) were obtained from MouseMine dataset (http://www.mousemine.org/mousemine/). Only single-gene transgenic mutant mouse strains were kept (*n* = 730), and these mouse genes were converted to their human orthologs (*n* = 728) based on data downloaded from the Mouse Genome Informatics (MGI) (ftp://ftp.informatics.jax.org/pub/reports/HOM_MouseHumanSequence.rpt). Mouse CHD genes were not split based into recessive/dominant because there was no concordance between autosomal dominant human CHD genes and mouse zygosity (of the 50 monoallelic human CHD genes with mouse models, only 20 have CHD observed on a heterozygous background).

Another set of genes used in this study is the top quarter of expressed genes during heart development (high heart expression, HHE genes), which was identified by RNA sequencing of mouse hearts at embryonic day E14.5 [1, 2].

### Statistical analysis

To identify potentially damaging mutations, we applied several filtering steps based on molecular class, allele frequency, intolerance to mutations, functional impact, and the number of variants in cases and controls. Here, it is important to note that the aim of this filtering strategy was to identify a set of variants that were highly likely to be pathogenic and the filtered-out variants were not necessarily benign.

The synonymous variants were filtered out from our analyses by giving priority to frameshift, nonsense, canonical splice site, start loss, missense, and non-frameshift insertion–deletion variants.

Functional variants with MAF < 0.001 across all samples in the Exome Aggregation Consortium (ExAC), the NHLBI Exome Sequencing Project (ESP), the Genome Aggregation Database (gnomAD), and the 1000 Genomes Project were examined by ANNOVAR [15–17, 23]. Variants whose frequency data were not available in any of the databases were also taken into consideration.

We evaluated intolerance of genes to mutations using the gene damage index (GDI) that provides an estimate for the accumulated mutational damage of each gene in the general population and helps to filter out highly damaged genes as those unlikely to be disease causing [24]. The genes with high GDI were filtered out from our dataset.

To improve the use of common variant-level methods that use a standard cut-off values across all genes, such as the Combined Annotation Dependent Depletion (CADD) score [25], we used the mutation significance cut-off (MSC) method with 95% confidence interval (CI) which provides gene-level and gene-specific low/high phenotypic impact cut-off values [26]. Since the variants with CADD≥MSC predicted scores suggest high functional effect, we filtered out the variants with CADD score below the MSC.

As a last step of filtration, the variants that were specific to the cases were determined by comparing the number of variants in cases to the number of variants in controls in each gene. Here, we tried several different approaches to decide how stringent a filter was appropriate for our data: (a) applying Fisher’s exact test on all genes, (b) applying Fisher’s exact test on only cases genes, (c) allowing all variants that are absent from controls, and (d) considering the genes in which *n*_cases_ − *n*_controls_ ≥ 2, where *n* is the number of variants. All approaches except for (d) did not show statistical significance in pathway analysis due to the small number of genes in cases that account for the likely genetic heterogeneity of CHD. Thus, we used (d) for the analyses described in this study.

Similar filtration steps, (i) removing synonymous, (ii) MAF < 0.001, (iii) low or medium GDI, (iv) CADD>MSC, and (v) *n*_controls_ − *n*_cases_ ≥ 2, were applied to the controls’ data.

DNVs occurring on X chromosome with X-linked recessive inheritance pattern were excluded from the analysis.

### Function, pathway, and network analysis

We investigated enrichment of variants in Gene Ontology (GO) terms and biological pathways using InnateDB, version 5.4 data analysis tool [27]. InnateDB performs a hypergeometric distribution test to find over-represented GO terms and pathways (imported from KEGG, NetPath, PID NCI, Reactome, INOH, and PID BioCarta) that are represented more than would be expected by random chance [28–33]. The NetworkAnalyst tool on String Interactome was applied with high-confidence (score > 0.9) to determine the interconnected subnetworks of protein-protein interactions (PPIs) [34, 35]. Additionally, Ingenuity Pathway Analysis (IPA) software, version 49309495 (http://www.qiagen.com/ingenuity) was used for identifying statistical significance of canonical pathways, diseases, biological functions, and networks that were most relevant to the input genes. To adjust the false discovery rate, the Benjamini-Hochberg (B-H) correction method was applied to the *p* values in all analyses. IPA analysis included the following parameters: (i) Ingenuity Knowledge Base (genes only) was used as the reference set, both direct and indirect relationships are considered; (ii) endogenous chemicals were included in networks interaction, the number of molecules per network was selected as 140, and the number of networks was selected as 25; (iii) all node types and all data sources were used; (iv) only experimentally observed information was considered; (v) molecules and interactions were limited to human only; (vi) molecules and relationships were selected from all tissues and cell lines; and (vii) all mutation findings were used.

### Biological distance calculations

The human gene connectome (HGC) is tailored to prioritize a given list of genes by their biological proximity to genes that are known to be associated with a phenotype of interest [36]. The biological proximity is defined by in silico predicted biologically plausible routes, distances, and degrees of separation between all pairs of human genes and calculated by a shortest distance algorithm on the full network of human protein-protein interactions. Since the causal genes of a specific phenotype are generally closely related via core genes or pathways, we determined the genes within the top 1% of each candidate gene’s connectome.

### Candidate gene prioritization

A priority score was defined to rank the genes based on their proximity to the known CHD-causing genes. For a given candidate gene, the score was the total number of known disease-causing genes in (i) the significantly enriched pathways (IPA canonical pathways, InnateDB pathways, GO terms); (ii) the networks (IPA network of cardiovascular diseases and PPI network); and (iii) the top 1% of genes connectome (significant proximity to the gene with *p* < 0.01) based on HGC. After ranking the candidate genes based on their priority scores, their expression levels during heart development were also taken into consideration.

To assess whether the known CHD-causing genes have higher priority scores as expected, we performed an independent two sample *t* test. We randomly selected 100 known CHD-causing genes and 100 genes from our filtered control set among the genes having more variants in controls than cases (*n*_controls_ > *n*_cases_), and compared the scores of two samples.

To test our gene candidates, we performed ToppGene suite and ranked the genes based on functional similarity to known CHD genes [37]. ToppGene first generated a representative profile from the training genes (known to be CHD-associated genes) based on functional properties such as gene expression, protein domains, protein interactions, gene ontologies, pathways, drug-disease associations, transcription factor-binding sites, and microRNAs, and then compared the candidate gene set to this profile. All available features were used with default test parameters. The genes were ranked based on their similarity to the known CHD-causing genes by calculating *p* values.

### Prediction of functional effects on proteins

Functional effects of amino acid substitutions were predicted using PROVEAN v1.1 that uses sequence alignment-based scoring and SNAP2 that is based on a variety of sequence and variant features [38, 39]. Both methods evaluate the effect of an amino acid substitution on protein function.

The PROVEAN score measures the change in sequence similarity of a given protein sequence to a protein sequence homolog before and after the variant occurs where the sequence similarity is computed by an amino acid substitution matrix. A score equal to or below a predefined threshold (default threshold = − 2.5) is considered to indicate a “deleterious” effect, and a score above the threshold is considered to indicate a “neutral” effect.

SNAP2 is a machine learning classifier based on a variety of sequence and variant features including the evolutionary information taken from multiple sequence alignment, secondary structure, and solvent accessibility. The predicted score ranges from −100 (strong neutral prediction) to +100 (strong effect prediction) and indicates the likelihood of variant to alter the protein function.

The intolerance of protein domains to functional variants was calculated using subRVIS [40]. SubRVIS calculates a rank for sub-regions of gene by their intolerance to functional variation. The sub-regions can be either defined as protein domains based on conserved domain sequences or exons. While a lower score indicates a more intolerant sub-region, a higher score indicates a more tolerant sub-region.

### Prediction of exonic splicing enhancers

We applied our in-house software to identify if the genetic variants were located in exonic splicing enhancers (ESEs) close to the canonical splice sites. There were in total 2341 ESE motifs collected from RESCUE-ESE, PESX, and SpliceAid [41–43]. By removing 16 duplicated ESEs from different resources, a collection of 2325 ESE motifs was retained for further analysis of our variants.

### Optimizing case-control ratio

Since the numbers of cases and controls were not equal (127 genes with 320 variants in cases and 36 genes with 73 variants in controls), we also tested our analysis on an extended control set. We randomly selected 91 genes from the 769 genes in controls where *n*_controls_ − *n*_cases_ = 1 and increased the size of the control set to 127 genes with 164 variants.

## Results

### Selection of de novo variants for analyses

We applied variant-level and gene-level filtrations on DNVs observed in 2645 CHD trios and 1789 controls. For the variant-level analysis, we filtered DNVs based on (i) functional effect, (ii) allele frequency, and (iii) phenotypic impact. For the gene-level, we filtered genes based on (i) accumulated mutational damage and (ii) the difference in the mutational burden between cases and controls (described in the “Methods” section). The results included 127 genes (320 variants) in cases and 36 genes (73 variants) in controls that we further explored in our analyses (Fig. 1a, b, Additional file 1: Tables S2 and S3). Notably, 232/320 variants were missense mutations (37 nonsense, 36 frameshift, 14 splicing mutations, and 1 start-loss) (Additional file 2: Figure S1). Among cases, 282 had only one predicted damaging DNV and 19 had two predicted damaging DNVs. In controls, 65 samples had only one predicted damaging DNV and four samples had two predicted damaging DNVs.

### Gene enrichment and pathway analyses

#### CHD DNVs are enriched in signaling pathways

In enrichment analyses, genes sets are tested for over-representation of shared biological or functional properties as defined by the reference databases; hence, the results depend on the database used in the analysis [44, 45]. As no single database covers all known pathway genes, a comprehensive interpretation of the results requires analyses be performed on several complementary databases. For example, while Ingenuity Pathway Analysis (IPA) software (QIAGEN Inc., https://www.qiagenbioinformatics.com/products/ingenuity- pathway-analysis) uses its own curated database, InnateDB uses major public databases (e.g., KEGG, Reactome) as resources [27, 28, 31]. Hence, to achieve a deeper understanding of the 127 genes in cases, we performed pathway analyses using both tools.

**Fig. 2:**
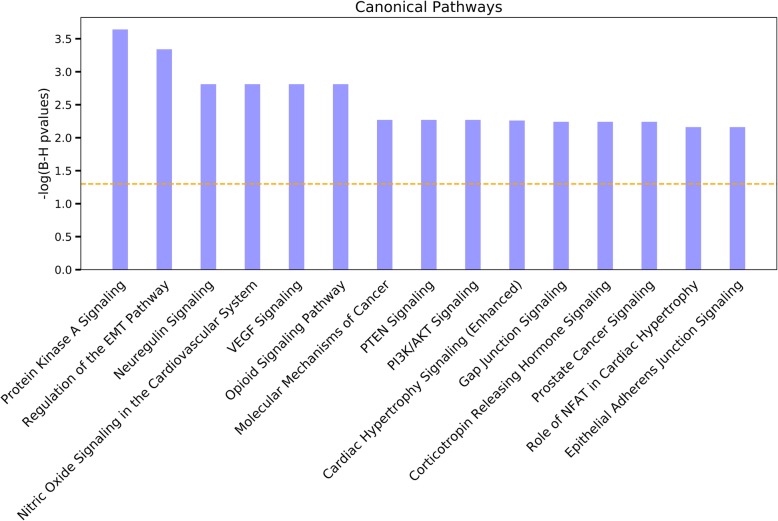
Top canonical pathways found in 127 genes in cases by the Ingenuity Pathway Analysis (IPA). Orange dashed line indicates the *p* value = 0.05 threshold. Only the top 15 pathways with *FDR* < 7 × 10^−3^ are shown. See Additional file 3: Table S4 for all data

We found 99 statistically significant canonical pathways (with a large proportion of biological overlap) by false discovery rate (FDR < 0.05) using IPA. The most significant pathways included the protein kinase A signaling (PKA) pathway, which is known to be associated with cardiac remodeling and arrhythmias [46, 47] (FDR = 2.29 × 10^−4^), regulation of the epithelial-mesenchymal transition (EMT), which plays crucial roles including for heart morphogenesis during development [48, 49] (FDR = 4.57 × 10^−4^), and nitric oxide signaling in the cardiovascular system [50] (FDR = 1.55 × 10^−3^) (Fig. 2, Additional file 3: Table S4). Overall, our results indicate significant enrichment of signaling pathways including Notch, ErbB and NF-κB signaling pathways that recent studies have associated with CHD [2, 3], as well as opioid, neuregulin, gap junction, VEGF, and FAK signaling pathways that were previously associated with heart disease [51–57].

The pathway analysis using InnateDB returned 211 over-represented pathways (with a large proportion of biological overlap) (FDR < 0.05), including VEGF, GPCR metabotropic glutamate receptor, PDGFR-beta, ERK, Notch, Igf, and NGF, affirming enrichment in signaling pathways (Additional file 3: Table S5). The most significant pathway was identified as focal adhesion (FDR = 1.72 × 10^−4^), which was found enriched by IPA as well and is known to have an important role in cellular differentiation and migration during cardiac development [56, 58, 59]. Another significantly enriched pathway was axon guidance (FDR = 0.0026). Slit-Robo signaling is known to have roles in axon guidance and has been suggested to be involved in heart development. Netrins, a class of axon guidance molecules, have also been suggested to have roles in cardiovascular biology and disease including angiogenesis [60–63].

Over-represented Gene Ontology (GO) terms included heart development (FDR = 8.96 × 10^−4^), axon guidance (FDR = 0.0011), pulmonary valve morphogenesis (FDR = 0.0018), chromatin binding (FDR = 0.0017), Notch signaling involved in heart development (FDR = 0.0035), histone-lysine-N-methyltransferase activity (FDR = 0.0035), and in utero embryonic development (FDR = 0.0053) (Additional file 3: Table S6). Histone-modifying genes and chromatin binding have been previously implicated to have a role in heart diseases [1, 64–66]. Interestingly, among the ten genes associated with the GO term heart development, only *CAD* had not been related to CHD previously.

#### No enrichment was detected in the extended control set

We did not identify any significant GO term or signaling pathway enriched in the control genes using IPA. By InnateDB, only five pathways had FDR < 0.05 (Additional file 3: Table S7). To check if the lack of enrichment in controls data could be attributable to smaller number of variants, we repeated all pathway enrichment analyses on an extended control set of the same size as for the cases, 127 genes with 164 DNVs (see the “Methods” section). Filtered DNVs in the extended control set did not show any significantly enriched canonical pathway by IPA. There were only one statistically significant Reactome pathway (FDR = 0.0027), transport of inorganic cations/anions and amino acids/oligopeptides, and no significant GO terms found by InnateDB in the extended control set. The lack of pathway enrichments in the controls group suggests the specificity of our results to CHD.

#### Enrichment in cardiovascular disease categories

To investigate the causal relatedness between the identified genes and biological functions/diseases, we analyzed the IPA-predicted top enriched diseases/functions categories (FDR < 0.05) and observed cardiovascular disease as a highly significant disease category in CHD cases (FDR = 5.36 × 10^−13^) (Additional file 3: Table S8). Among the disease subcategories under “cardiovascular disease” category, familial cardiovascular disease was the most enriched. As the biological function/disease categories have a hierarchical nature, the following enriched cardiovascular disease subcategories give more specific information on candidate genes. For example, while *CDK13*, *CHD4*, *KDM5A*, and *SCN10A* are related to familial heart disease, *CFH*, *DGUOK*, and *POLE* are related to familial vascular disease. In contrast, the only statistically significant cardiovascular disease in controls was the branching morphogenesis of vascular endothelial cells with FDR = 0.013, and involved only the gene *PTPRJ.* Taken together, these results suggest that the candidate CHD genes are enriched in phenotypes that are closely associated with CHD.

#### A high-confidence subnetwork associated with cardiovascular disease

**Fig. 3:**
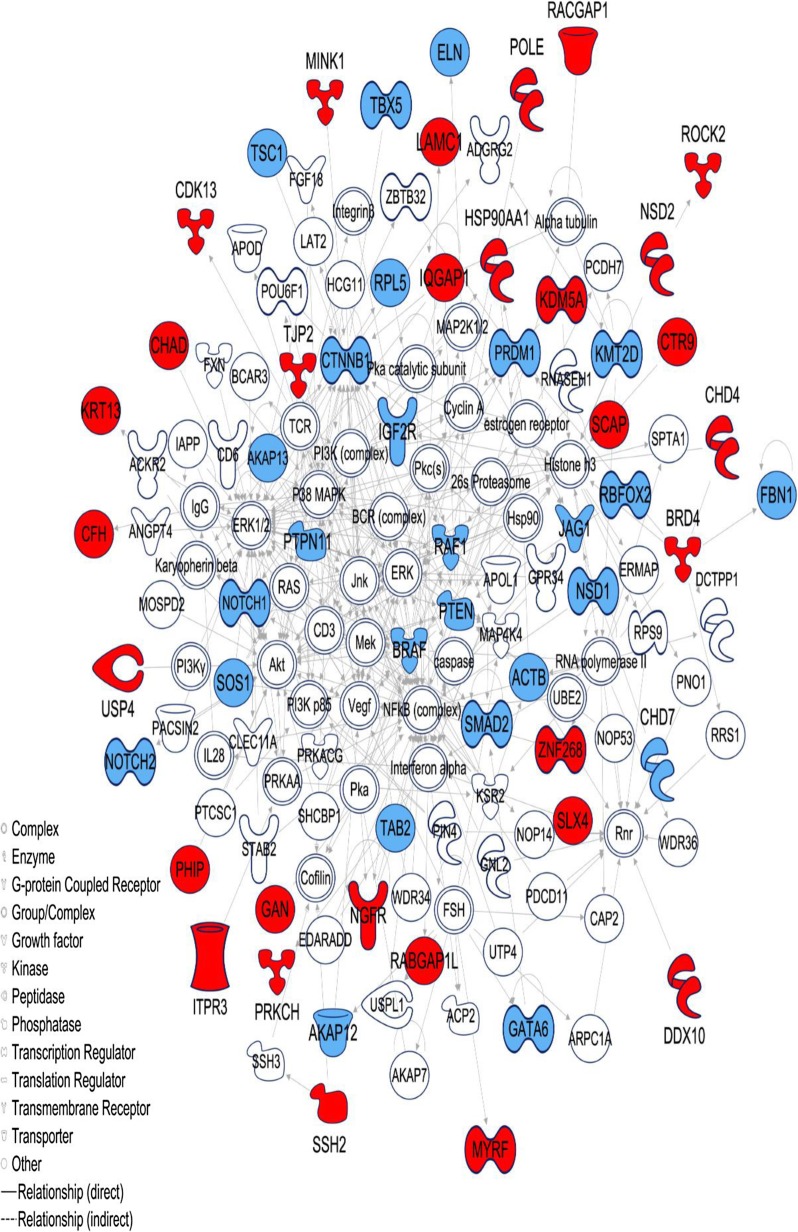
Subnetwork in cases associated with “Cardiovascular Disease, Hereditary Disorder, Organismal Injury and Abnormalities”. Generated by IPA software. Network includes 140 nodes with 56 input genes where 26 known CHD-causing genes are shown in blue and 30 likely CHD-associated genes are shown in red color

In addition to pathways that describe a specific biological function and gene ontologies that describe gene functions, we also analyzed biological networks that describe biological relationships and interactions between biomolecules to further explore significant gene-CHD associations. IPA identified two significant subnetworks for cases with *p* values of 10^−85^ and 10^−45^, indicating enrichment of interactions and biological relatedness between the genes (Additional file 3: Table S9a). The network with the most significant *p* value (*p*=10^−85^) included 56 genes from our input gene set (or “focus genes” as defined by the IPA), and interestingly, the network genes were found associated with cardiovascular disease, hereditary disorder, and organismal injury and abnormalities (Fig. 3). Notably, 26/56 genes are known CHD-causing genes (*p* < 10^−4^ by chi-squared test, comparing to 187/2504 in all cases genes). The second significant subnetwork (*p* = 10^−45^) included 36 focus genes and was associated with cancer, cellular development, and cellular growth and proliferation disorders. In controls, the most significant subnetwork (*p* = 10^−38^) included 22 focus genes and was associated with cell death and survival, cellular movement, and connective tissue development and function disorders. We did not identify a network related to cardiovascular disease among the control genes or in the extended control set.

**Fig. 4:**
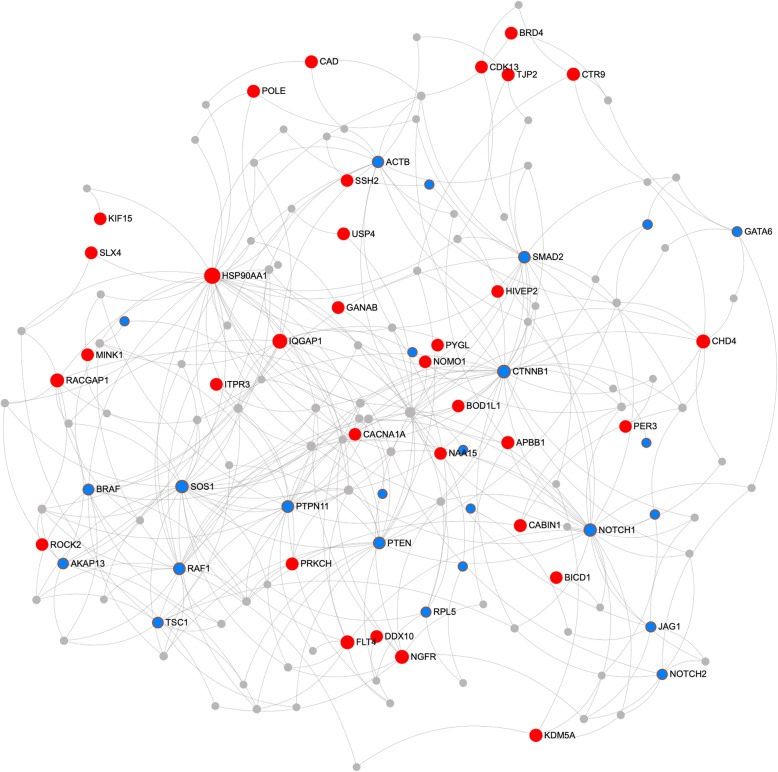
Protein-Protein interaction network generated by String interactome with medium (400) to high (1000) confidence score using NetworkAnalyst web tool. Network includes 149 nodes with 58 input genes where the known CHD-causing genes are shown in blue and likely CHD-associated genes are shown in red color

We also generated a protein-protein interaction network by the NetworkAnalyst tool on the String Interactome (Additional file 3: Table S9b) to verify our results and determined a subnetwork of 149 genes including 58 input genes using the minimum network option with *P* = 2.5 × 10^−5^ [34, 35] (Fig. 4). Despite the fact that this network was generated based only on direct protein-protein interactions (PPIs), unlike the IPA network for which both direct and indirect interactions between all biomolecules are considered, there was a large overlap between the two networks (39 common genes). Furthermore, the most significant GO biological process term found in this subnetwork was heart development (FDR = 5.51 × 10^−10^), followed by circulatory system development (FDR = 1.71 × 10^−7^) [34]. Considering all of these findings, we suggest that involvement in a network associated with cardiac disease with a significant number of interactions supports the role of candidate network genes in CHD.

#### Validation of the enrichment results in cases

To assess our findings in the cases, we repeated our analysis on an independent CHD cohort comprising 559 parent-offspring trios with a total of 977 de novo variants. After following the same variant filtering method that we applied on cases and controls (described in the “Methods” section), we identified 30 genes (with 54 DNVs) to further analyze (Additional file 4: Table S10). Despite the smaller sample size, we again observed enrichment in signaling pathways including opioid, netrin, protein kinase A, and axonal guidance, as well as enrichment in GO terms including blood vessel development and embryonic heart tube development (Additional file 4: Tables S11-S13). The most significant network identified by IPA (*p* = 10^−54^) included 26 genes and was associated with cardiac dysfunction, cardiovascular disease, and organismal injury and abnormalities (Additional file 4: Table S14a). We further explored our findings by randomly selecting 30 genes from the unfiltered dataset of 559 samples and repeating the enrichment analyses. In the random set of genes, we did not identify any significantly enriched pathway, or a network related to cardiovascular disease. There were only some GO terms with FDR > 0.04 including a single gene, which were not significantly enriched in the cases (Additional file 4: Table S15). These results validated that our approach is effective in identifying CHD-related gene pathways and networks.

### Candidate novel CHD-causing genes

Our gene enrichment analysis results revealed that some genes that were not among currently known CHD-causing genes (see the “Methods” section) were involved in multiple significantly enriched pathways and in a network of cardiovascular disease together with known CHD-causing genes. Since we have applied relaxed criteria to allow analyses of additional genes, these genes had a low number of hits (2 or 3), while the genes with higher number of hits (> 5) were all known genes (*KMT2D*: 16, *CHD7*: 15, *PTPN11*: 10, and *NOTCH1*: 6) (Additional file 5: Table S16). To identify the most plausible novel CHD-causing gene candidates, we performed systematic analyses by considering involvement in enriched pathways, connections in the biological networks, and expression levels during heart development.

#### Gene prioritization

To assess novel candidate CHD-causing genes suggested by the enrichment analyses in the previous section, we defined a priority score (see the “Methods” section), where a higher score indicates the gene’s connectivity to a high number of known CHD-causing genes through (i) multiple significant pathways (FDR < 0.05) [27–33, 67], (ii) multiple significant networks [34, 67, 68], and (iii) the Human Gene Connectome (HGC) [36]. We also checked if the candidate gene was highly expressed during heart development (Additional file 5: Table S16) [1, 2]. Pathway and network analysis have been effectively integrated in candidate gene prioritization by different methods based on the rationale that disease-associated genes/proteins interact with each other [69–71]. Similarly, the biological distance between candidate genes and known disease-causing genes is shown to be an efficient measure for gene prioritization [72]. Altogether, these analyses that are based on different heterogenous data types and data sets provided partially overlapping and complementary information, resulting in prioritizing the plausible candidate genes based on the combined evidence of their biological relatedness to the known CHD-causing genes.

**Fig. 5:**
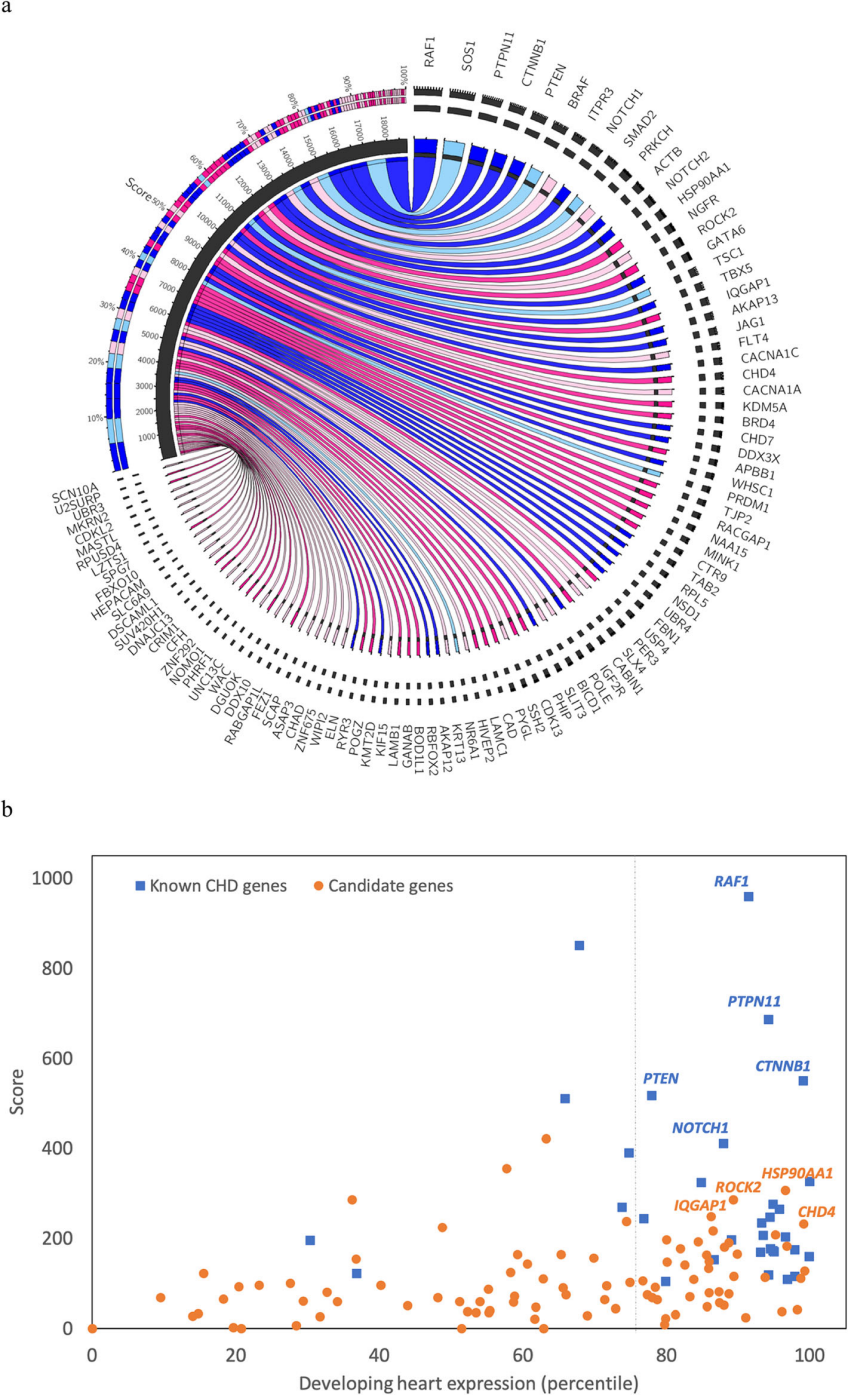
Priority score and expression level during heart development of genes in cases. **a** Circos map illustrating the top 100 genes among 127 filtered genes in cases. The genes are ordered in clockwise direction with respect to the scores. The known CHD-associated genes are indicated by color blue, and the candidate genes are indicated by color pink. Different shades of colors indicate expression level during heart development (darker shade indicates high expression). The inner ring in the score segment represents the score of each gene, and the outer ring represents the relative contribution of each gene’s score to the total score. **b** All 95 candidate genes and 32 known CHD genes, where the *x*-axis (0–100) denotes the percentile of heart expression in developing (E14.5) mouse heart, and the *y*-axis denotes the priority score. The candidate genes are shown in blue squares, and known CHD-causing genes are shown in orange circles. The high scored genes that are in the top 25% of expression in developing heart, *HSP90AA1*, *ROCK2*, *IQGAP1*, and *CHD4*, are selected as the most plausible gene candidates

Among all 127 case genes that we identified, 95 were not previously associated with CHD and 41 of them were also highly expressed during heart development. The Circos plot [73] of genes in cases with respect to the scores is shown in Fig. 5a. The 32 known CHD-causing genes had scores ranging between 105 and 960. Among the 95 CHD-causing candidate genes, 38 had scores ranging between 109 and 422, falling into the same range of the scores of known CHD-causing genes. To test our scoring method, we performed an independent samples *t* test to compare scores of 100 randomly selected known CHD-causing genes and 100 randomly selected control genes. The 95th percentile confidence intervals for the scores of the CHD-causing and control genes were 443–608 and 20–25, respectively. There was a significant difference in the scores of known CHD genes (mean = 525.59, sd = 421.5) and scores of controls (mean = 22.54, sd = 11.6); *t* = 11.86, *p* = 9.55 × 10^−21^. Among the 38 candidate genes, 23 were highly expressed in embryonic mouse heart [1] (Fig. 5b), adding to their plausibility. The genes *HSP90AA1*, *ROCK2*, *IQGAP1*, and *CHD4* were on the top of the list. Among 23 candidate genes, damaging DNVs in nine of them (*ROCK2*, *CHD4*, *KDM5A*, *APBB1*, *USP4*, *PYGL*, *CAD*, *BOD1L1*, and *GANAB*) were found in syndromic CHD patients, in three of them (*HSP90AA1*, *IQGAP1*, and *TJP2*) were found in isolated CHD patients, and remaining were found in either both types of CHD or those with unknown phenotype status (Additional file 5: Table S16). Interestingly, there were two loss-of-function heterozygous mutations in *HSP90AA1* as reported in the previous study on the same cohort [9]. Additional file 2: Figure S2 shows the phylogenic tree based on HGC biological distances between the 95 novel candidate genes and 32 known CHD-causing genes among the 127 filtered case genes. Most of the candidate genes with high scores were scattered among the branches of known CHD-causing genes, while the genes with low score were clustered as an outgroup, further supporting the plausibility for functional relevance of these candidate genes to CHD.

To investigate if considering mouse CHD genes as known CHD-causing genes had an impact on our results, we repeated our analysis with only human CHD genes as the known genes. All novel candidate genes were again ranked at top of the list along with nine mouse CHD genes (see Additional file 5: Table S17). We further calculated the average biological distance of candidate genes with respect to human CHD genes only (mean = 13.36, sd = 4.27) and mouse CHD genes only (mean = 13.04, sd = 4.17). The average distances showed no significant difference (independent *t* test, *t* = 0.57, *p* = 0.56) when using human or mouse CHD genes (Additional file 5: Table S18), supporting the notion that mouse CHD genes were plausible to use in this study.

#### Tissue enrichment in candidate genes

We examined the expression of 23 novel candidate genes using the Human Protein Atlas (HPA) RNA-seq data and observed that 20/23 of the genes were expressed in all tissues or mixed, and 3/23 were tissue enhanced (*LAMB1*: placenta, *LAMC1*: placenta, and *RACGAP1*: testis). We also observed that the majority of the known CHD-causing genes (67.5%) are expressed in all or mixed and the rest (32.5%) have elevated expression (tissue enhanced/enriched or group enriched), while approximately 54% of the protein coding genes in human body is expressed in all/mixed [74, 75] (https://www.proteinatlas.org/). While the tissue expression profiles of the candidate genes are significantly different from expression levels of all genes (chi-square with Yates correction, two-tailed *p* value = 0.0077), there is no significant difference from the expression profiles of the known CHD-causing genes (chi-square with Yates correction, two-tailed *p* value = 0.08).

#### Association of candidate genes with known CHD-causing genes

We used the Human Gene Connectome (HGC) server to calculate the distances of candidate genes to the known CHD-causing genes [36, 76]. The HGC provides biological/functional distance between any two human genes, which is defined as the weighted sum of direct distances in the shortest path connecting the two genes. Table 1 presents the closest known CHD-causing gene and its route to the candidate genes. The *p* values indicated that 20 of the candidate genes are in the first percentile of the corresponding known gene’s connectome.

**Table 1:** The closest known CHD-causing gene to the 23 candidate genes calculated by HGC

Candidate	Known	Distance	*p* value	Route	Degrees of separation
APBB1	MGRN1	1.77	0.00078	MGRN1[1.77]APBB1	1
BOD1L1	SOX2	3.27	0.01344	SOX2[3.26]BOD1L1	1
BRD4	HEXIM1	1.25	0.00012	HEXIM1[1.25]BRD4	1
CABIN1	PPP3CB	1.11	0.00239	PPP3CB[1.11]CABIN1	1
CAD	ACACB	1.04	0.00012	ACACB[1.04]CAD	1
CDK13	CDC73	1.48	0.00227	CDC73[1.48]CDK13	1
CHD4	ZEB2	1.25	6.00E-05	ZEB2[1.25]CHD4	1
CTR9	CDC73	1.08	0.00024	CDC73[1.07]CTR9	1
GANAB	CALR	1.11	0.0003	CALR[1.11]GANAB	1
HSP90AA1	AIP	1.11	6.00E-05	AIP[1.11]HSP90AA1	1
IQGAP1	WHSC1	1.25	0.0003	WHSC1[1.25]IQGAP1	1
KDM5A	RBPJ	1.25	0.00329	RBPJ[1.25]KDM5A	1
LAMB1	DAG1	1.11	0.00054	DAG1[1.11]LAMB1	1
LAMC1	DAG1	1.11	0.00167	DAG1[1.11]LAMC1	1
MINK1	KIFAP3	1.75	0.0009	KIFAP3[1.74]MINK1	1
NAA15	NEK1	6.01	0.01703	NEK1[1.25]XRCC5[1.75]NAA15	2
PHIP	CSNK2A1	3.28	0.01285	CSNK2A1[3.27]PHIP	1
POGZ	HDAC7	4.08	0.00759	HDAC7[4.08]POGZ	1
PYGL	GBE1	4.44	0.00191	GBE1[1.11]GYG2[1.11]PYGL	2
RACGAP1	DSP	4.72	0.00269	DSP[1.25]PKP4[1.11]RACGAP1	2
ROCK2	MYH10	1.11	0.00036	MYH10[1.11]ROCK2	1
TJP2	RAPGEF2	1.25	0.00018	RAPGEF2[1.25]TJP2	1
USP4	HUWE1	1.77	0.00149	HUWE1[1.77]USP4	1

#### Assessing candidate genes with ToppGene

To further validate our findings, we also prioritized genes based on their functional similarity to the known genes by using ToppGene suite [37]. Ten of the 23 novel candidate genes were also ranked at the top by ToppGene with *p* < 10^−3^ (Additional file 5: Table S16). The ranked gene list was in good agreement with our list of candidate genes.

#### Candidate genes in isolated and syndromic CHD

Among 301 CHD cases carrying possibly damaging DNVs, 73 were isolated CHD patients (CHD without extracardiac manifestation or neurodevelopmental deficiency) and 180 were syndromic CHD patients (with EM and/or NDD) (Additional file 1: Table S2). To investigate the pathways and genes altered in these two different types of CHD, we performed pathway enrichment analyses and gene prioritization in the two subgroups separately. We identified 64 candidate genes involved in isolated CHD and 105 candidate genes involved in syndromic CHD (45 involved in both). In isolated CHD, the pathways including nitric oxide signaling in the cardiovascular system, PKA signaling, Igf receptor activity, positive regulation of cardioblast differentiation, Notch signaling involved in heart development, and pulmonary valve morphogenesis were found to be highly enriched (Additional file 6: Tables S19–21). Some of these pathways (e.g., Notch1, Igf-1 signaling) were reported in a recent study of Sifrim et al. on a predominantly nonsyndromic CHD cohort [3]. In syndromic CHD, the pathways such as PKA signaling, opioid signaling, heart development, chromatin binding, and focal adhesion were found to be significantly enriched (Additional file 6: Tables S24–26). Despite the smaller sample sizes, following our gene prioritization approach, we identified 11 and 22 candidate genes for isolated and syndromic CHD, respectively (Additional file 6: Tables S23 and S28). Top candidate genes in isolated CHD include *HSP90AA1*, *IQGAP1*, and *TJP2*, and top candidate genes in syndromic CHD include *ROCK2*, *APBB1*, *KDM5A*, and *CHD4*.

#### Candidate genes in patients with conotruncal defects and left ventricular obstruction

Cardiac phenotypes of the CHD proband were defined as (i) conotruncal defects (CTD, 30%), (ii) d-transposition of the great arteries (d-TGA, 9%), (iii) heterotaxy (HTX, 9%), (iv) left ventricular outflow tract obstruction (LVO, 28%), and (v) other (24%) in the previously reported study [9] (see Additional file 2: Figure S3 for details). Among 301 patients carrying possibly damaging DNVs, 84 had CTD (27.5%), 21 had d-TGA (7%), 23 had HTX (7.5%), 99 had LVO (33%), and 74 had other (25%) types of CHD (Additional file 1: Table S2). We identified 59 candidate genes in CTD and 68 candidate genes in LVO and, therefore, were able to perform a subgroup analysis for these two subtypes of CHD. Pathway analyses in CTD genes showed that VEGF signaling, PKA signaling, axon guidance, distal tube development, and Igf-1 signaling pathways were highly enriched (Additional file 7: Tables S29–31). After prioritizing the genes, *ROCK2* was on top of the list (Additional file 7: Table S33). LVO genes showed significant enrichment in CDK5 signaling, Notch signaling, pulmonary valve morphogenesis, and Beta3 integrin cell surface interactions pathways (Additional file 7: Tables S34–36). Gene prioritization revealed that the top genes include *KDM5A* and *PHIP* (Additional file 7: Table S38).

### Function-affecting genetic variants in candidate CHD-causing genes

To verify that the 23 novel candidate genes were unlikely to be false positives, we checked if the variants in those genes existed in the non-pathogenic genetic variants list, the “blacklist” [66]. This recently curated list includes variants absent or rare in public databases but too common in patients suffering from severe genetic diseases and, therefore, are unlikely to cause disease. None of our damaging DNVs was included in the blacklist.

Next, to evaluate whether the 41 missense variants in the 23 strong candidate genes are likely to have functional effects, we analyzed them with PROVEAN and SNAP2 [38, 39] (Additional file 8: Table S39). We did not use the functional impact prediction tools in the filtering step as we considered all non-synonymous mutations, and they provide a score for missense mutations only. Among 41 missense variants, 24 were predicted to be damaging by both tools and 6 were predicted to be damaging by one of the tools. We also estimated the intolerance of protein domains to functional variation using the subRVIS [40] tool to further analyze the effects of the DNVs in candidate CHD-causing genes. Among 41 variants, 31 were found to affect regions intolerant to mutations and, therefore, more likely to cause disease. We then checked if the candidate CHD-causing genetic variants were already included in the HGMD database [77]. Four DNVs (one in *CDK13*, one in *KDM5A*, and 2 in *NAA15*) were classified as CHD-causing variants, and 23 DNVs were classified as likely to be CHD-causing mutations in the HGMD Professional 2019.2 database (Additional file 8: Table S39).

To check the population genetics-level functional impact of the variants occurring in the top four candidate genes (*HSP90AA1*, *ROCK2*, *IQGAP1*, and *CHD4*), we visualized the minor allele frequencies with respect to damage prediction scores (CADD) using PopViz [78]. Additional file 2: Figure S4 displays all missense variants in European population with CADD>MSC score (95% confidence interval) in gnomAD database [23]. These plots suggest that the rare variants in the top candidate genes likely have a strong functional impact.

Interestingly, five of the 23 candidate genes (*ROCK2*, *BRD4*, *TJP2*, *MINK1*, and *CDK13*) were kinases (Table 2), a class of proteins that has previously been implicated in cardiac diseases [79–83]. Two of the DNVs, p.D255G in *ROCK2* and p.N842S in *CDK13*, were predicted to alter the protein kinase domains by subRVIS [40] (Additional file 8: Table S39). Mutations in the kinase domain of *CDK13* were previously found to be related to a syndromic form of intellectual disability with or without congenital heart disease [84].

**Table 2:** Twenty-three plausible CHD candidate genes

Gene ID	Gene name	Type
APBB1	Amyloid beta precursor protein binding family B member 1	Transcription regulator
BOD1L1	Biorientation of chromosomes in cell division 1 like 1	Other
BRD4	Bromodomain containing 4	Kinase
CABIN1	Calcineurin binding protein 1	Other
CAD	Carbamoyl-phosphate synthetase 2, aspartate transcarbamylase, and dihydroorotase	Enzyme
CDK13	Cyclin dependent kinase 13	Kinase
CHD4	Chromodomain helicase DNA binding protein 4	Enzyme
CTR9	CTR9 homolog, Paf1/RNA polymerase II complex component	Other
GANAB	Glucosidase II alpha subunit	Enzyme
HSP90AA1	Heat shock protein 90 alpha family class A member 1	Enzyme
IQGAP1	IQ motif containing GTPase activating protein 1	Other
KDM5A	Lysine demethylase 5A	Transcription regulator
LAMB1	Laminin subunit beta 1	Other
LAMC1	Laminin subunit gamma 1	Other
MINK1	Misshapen like kinase 1	Kinase
NAA15	*N* (alpha)-Acetyltransferase 15, NatA auxiliary subunit	Transcription regulator
PHIP	Pleckstrin homology domain interacting protein	Other
POGZ	Pogo transposable element derived with ZNF domain	Enzyme
PYGL	Glycogen phosphorylase L	Enzyme
RACGAP1	Rac GTPase activating protein 1	Transporter
ROCK2	Rho-associated coiled-coil containing protein kinase 2	Kinase
TJP2	Tight junction protein 2	Kinase
USP4	Ubiquitin specific peptidase 4	Peptidase

#### Synonymous DNVs in exonic splicing enhancers

To check if synonymous DNVs in cases contribute to CHD, we analyzed them by first applying the same filtering steps as described for the other variant types, and next performing enrichment analyses. We identified nine genes having two synonymous variants in cases and none in controls. Four of these genes (*HSP90B1*, *GIT1*, *ARID1B*, and *CASZ1*) were highly expressed during heart development. Interestingly, one of these genes, *HSP90B1*, was previously associated with CHD. We applied the state-of-the-art pathogenicity prediction tool, S-CAP, and calculated scores of eight synonymous variants [85]. Except for the two synonymous variants in *CASZ1*, all six variants were predicted to be pathogenic by S-CAP. We further applied our in-house software to identify if these variants are located in the exonic splicing enhancers (ESE) near the canonical splice sites (see the “Methods” section). We observed the variant (chr12-104336346-C-T), which locates + 41 bp of the splice acceptor site of exon 12 of gene *HSP90B1*, was shown to overlap with 7 aligned ESE motifs (GATCAA, ATCAAG, CAAGAA, TCAAGA, CAAGAAGA, TCAAGAAG, ATCAAGAA). The underscored nucleotide in each motif sequence is where the variation occurs. These seven ESE motifs are aligned to the same genomic region close to the splice acceptor site, suggesting the importance of this region to bind with SR proteins to promote the exon splicing. The variant changes the highly conserved C to T in these ESE motifs, which may result in reduced or inhibited affinity for splicing factors. Subsequently, the altered ESEs by this variant may in turn lead to the aberrant splicing events.

## Discussion

Here, we performed a comprehensive analysis of DNVs in a large set of CHD patient and control trio data. Our goal was to identify novel CHD-associated candidate genes through pathway/network analyses and by using the controls and a validation set to assess the significance of our findings. Our approach included variant filtering to identify potentially damaging DNVs followed by enrichment analysis and knowledge-driven prioritization based on biological pathways, annotations, molecular interactions, functional similarities, and expression profiles. While filtering and prioritization depend on the specific study at hand, we demonstrate that our procedure yielded plausible candidate genes with statistically significant enrichment by supporting evidence from multiple aspects.

Unlike previous CHD studies where gene-level case-control studies were performed, in this study, we applied a pathway-level approach to identify risk genes. Another major novel component of our analysis was comparing the number of variants in cases and controls instead of applying a strict gene burden filter such as Fisher’s exact test. To account for the very low number of hits in individual genes, we followed a relaxed approach, thereby obtaining sufficient numbers of potentially disease-causing mutations to enable statistical power for case-control enrichment analyses.

Pathway analysis showed significant enrichment in heart development and signaling pathways (i.e., PKA, EMT, nitric oxide signaling, focal adhesion) in filtered cases genes that have been previously associated with heart disease, and conversely, no enrichment was found in filtered controls genes [3, 9]. In addition to previously known CHD-associated genes, we also observed novel genes involved in these pathways. Since we have applied a relaxed approach to include more candidate genes into pathway analyses, we evaluated the plausibility of each candidate gene.

To prioritize the candidate genes, we defined a priority score based on the number of known CHD-causing genes in a candidate gene’s pathway, network, and HGC distance to known CHD-causing genes. The higher scores and high expression levels during heart development provided supporting evidence for candidate genes, since a majority (54%) of human CHD genes are highly expressed in the developing heart. It is also important to note that the genes with lower scores or lower expression levels should be considered as candidates with less evidence. The genes *HSP90AA1*, *ROCK2*, *IQGAP1*, and *CHD4* were at the top of the list with highest scores and as being highly expressed during heart development. For example, *HSP90AA1* is associated with pathways including nitric oxide signaling in the cardiovascular system, VEGF signaling that has been shown to be linked to CHD [86–88], and axon guidance; *ROCK2* is associated with pathways including PAK signaling, VEGF signaling, focal adhesion, and axon guidance; *IQGAP1* is associated with IL-8 signaling, epithelial adherens junction signaling, and EGFR1; and *CHD4* is associated with Th2 pathway, transcription factor binding, and zinc ion binding.

Notably, DNVs in *HSP90AA1* and *IQGAP1* were found in isolated CHD patients, whereas DNVs in *ROCK2* and *CHD4* were found in syndromic CHD patients. Two DNVs in *CHD4* (p.Y1345D and p.M202I), p.R1330W in *IQGAP1*, and p.S39F in *ROCK2* were previously associated with CHD and p.M954I in *CHD4* was associated with developmental disorder [2, 3, 9] (Additional file 8: Table S39). Overall, our findings suggested 23 novel plausible genes contributing to CHD.

To ensure that our results were robust and not biased as a result of lower number of filtered control variants compared to cases (320 variants in cases and 73 variants in controls), we repeated our analyses on an extended control set. We still did not identify any significant enrichment in the extended control gene set.

To test our filtering strategy, we also performed enrichment analysis on rare DNVs after removing the synonymous variants (2278 variants in 1951 genes) without further filtering. Significant enrichment persisted in signaling pathways and cardiovascular diseases among 1951 genes supporting our findings for potentially damaging DNVs.

Due to the extreme heterogeneity of CHD, gene-level approaches have statistical power limitations for suggesting novel risk genes. This study represents a pathway-level approach that enables discovery of novel plausible CHD risk genes. We considered all genes having at least two more DNVs in cases than controls to be able to reach pathway-level statistical significance. However, it is important to note that this criterion depends on the size of the cohort and characteristic of the disease. While this approach has been efficient for identifying novel risk genes in this large cohort, we anticipate that it can be applied for studying rare variants in other genetically heterogeneous diseases.

## Conclusions

Previous approaches that use DNVs to estimate variant rates or perform gene-level case-control analysis have limitation on identifying novel CHD genes due to extreme genetic heterogeneity of the disease. A recent study comparing the observed and expected rates of DNVs on the same data suggested 66 genes having more than one damaging de novo variants as risk genes [9]. Among those, only five genes (*CHD7*, *KMT2D*, *PTPN11*, *GATA6*, and *RBFOX2*) reached genome-wide significance and all were already known CHD-causing genes. In this study, we aimed to discover new plausible candidate genes and applied a pathway-level approach that enabled us to discover 23 novel genes. Our approach explored whether genes having a low number of hits altered common molecular pathways in CHD patients and prioritized genes based on their biological proximity to the known CHD-causing genes. This large-scale study indicates that using pathway-level approaches is effective to analyze the effects of rare de novo variants in heterogenic diseases.

## Supplementary information

**Additional file 1: **Cases and controls: **Table S1.** Ethnicity and sex distributions of all CHD cases and controls, **Table S2.** Filtered de novo mutations in cases, **Table S3.** Filtered de novo mutations in controls.

**Additional file 2: Figure S1.** Variant types of filtered 320 DNVs in cases, **Figure S2.** A phylogenetic tree of biological distances between 32 known CHD-causing genes and 95 candidate genes in cases, **Figure S3.** Cardiac phenotypes considered in this study, **Figure S4.** Minor allele frequency of all missense mutations in: A) *HSP90AA1*, B) *ROCK2*, C) *IQGAP1*, D) *CHD4*.

**Additional file 3: **Enrichment analyses in cases and controls: **Table S4.** IPA canonical pathways among 127 genes in cases, **Table S5.** InnateDB pathway analysis among 127 genes in cases, **Table S6.** InnateDB GO analysis among 127 genes in cases, **Table S7.** InnateDB pathway analysis among 36 genes in controls, **Table S8.** IPA top diseases and functions among 127 genes in cases, **Table S9a.** IPA Networks among 127 genes in cases, **Table S9b.** NetworkAnalyst PPI Network among 127 genes in cases.

**Additional file 4: **Enrichment analyses in validation set: **Table S10.** De novo mutations in the filtered validation dataset, **Table S11.** IPA canonical pathways among 30 genes in validation dataset, **Table S12.** InnateDB pathway analysis among 30 genes in validation dataset, **Table S13.** InnateDB GO analysis among 30 genes in validation dataset, Table S14a. IPA Networks among 30 genes in validation dataset, **Table S14b.** NetworkAnalyst PPI Network among 30 genes in validation dataset, **Table S15.** InnateDB GO analysis among 30 random genes from unfiltered validation dataset.

**Additional file 5: **Gene prioritization: **Table S16.** Priority scores of 127 genes in cases, **Table S17.** Priority scores of 127 genes in CHD cases based on human CHD genes only, **Table S18.** Average biological distance of each candidate gene to human and mouse CHD genes based on Human Gene Connectome.

**Additional file 6: **Enrichment analysis in isolated and syndromic CHD: **Table S19.** IPA canonical pathways among 64 genes in isolated CHD cases, **Table S20.** InnateDB pathway analysis among 64 genes in isolated CHD cases, **Table S21.** InnateDB GO analysis among 64 genes in isolated CHD cases, **Table S22.** NetworkAnalyst PPI Network among 64 genes in isolated CHD cases, **Table S23.** Priority scores of 64 genes in isolated CHD cases, **Table S24.** IPA canonical pathways among 105 genes in syndromic CHD cases, **Table S25.** InnateDB pathway analysis among 105 genes in syndromic CHD cases, **Table S26.** InnateDB GO analysis among 105 genes in syndromic CHD cases, **Table S27.** NetworkAnalyst PPI Network among 105 genes in syndromic CHD cases, **Table S28.** Priority scores of 105 genes in syndromic CHD cases.

**Additional file 7: **Enrichment analysis in CTD and LVO: **Table S29.** IPA canonical pathways among 59 genes in conotruncal defects (CTD), **Table S30.** InnateDB pathway analysis among 59 genes in conotruncal defects (CTD), **Table S31.** InnateDB GO analysis among 59 genes in conotruncal defects (CTD), **Table S32.** NetworkAnalyst PPI Network among 59 genes in conotruncal defects (CTD), **Table S33.** Priority scores of 59 genes in conotruncal defects (CTD), **Table S34.** IPA canonical pathways among 68 genes in left ventricular outflow tract obstruction (LVO), **Table S35.** InnateDB pathway analysis among 68 genes in left ventricular outflow tract obstruction (LVO), **Table S36.** InnateDB GO analysis among 68 genes in left ventricular outflow tract obstruction (LVO), **Table S37.** NetworkAnalyst PPI Network among 68 genes in left ventricular outflow tract obstruction (LVO), **Table S38.** Priority scores of 68 genes in left ventricular outflow tract obstruction (LVO).

**Additional file 8: Table S39.** Functional effects of DNMs on 23 plausible candidate genes in cases, **Table S40.** De novo mutations in validation set, **Table S41.** Human CHD genes, **Table S42.** Mouse CHD genes.

## Data Availability

Cases’ and controls’ datasets used in this study are available in the supplementary datasets (Tables S9 and S10) in the previously published paper 10.1038/ng.3970 [9]. Validation dataset is included in Additional file 8: Table S40. Known CHD gene sets are available in Additional file 8: Tables S41-S42. Expression percentiles of genes in the developing mouse heart are available in Additional file 4: Table S10 of [2]. The code for the in-house script used for predicting the genomic variants affecting exonic splicing enhancers (ESEs) is available by request from the corresponding author. All data generated or analyzed during this study are included in the supplementary files.

## Abbreviations

B-H: Benjamini-Hochberg
CADD: Combined Annotation Dependent Depletion
CHD: Congenital heart disease
CI: Confidence interval
DNV: De novo variant
EMT: Epithelial-mesenchymal transition
ESE: Exonic splicing enhancer
ESP: Exome Sequencing Project
ExAC: Exome Aggregation Consortium
FDR: False discovery rate
GDI: Gene damage index
gnomAD: The Genome Aggregation Database
GO: Gene Ontology
HGC: The Human Gene Connectome
HHE: High heart expression
HPA: Human Protein Atlas
IBD: Identity-by-descent
IPA: Ingenuity Pathway Analysis
MAF: Minor allele frequency
MGI: Mouse Genome Informatics
MPO: Mammalian Phenotype Ontology
MSC: Mutation significance cut-off
PCGC: Pediatric Cardiac Genetics Consortium
PHN: Pediatric Heart Network
PKA: Protein kinase A signaling
PPI: Protein-protein interaction
WES: Whole-exome sequencing
