# Supplementary material for: De novo variants in exomes of congenital heart disease patients identify risk genes and pathways
Source: Genome Med. 2020 Jan 15;12:9. doi: 10.1186/s13073-019-0709-8 (PMC6961332; doi:10.1186/s13073-019-0709-8)
Supplement: Supplementary file 2 — Additional file 2: Figure S1. Variant types of filtered 320 DNVs in cases, Figure S2. A phylogenetic tree of biological distances between 32 known CHD-causing genes and 95 candidate genes in cases, Figure S3. Cardiac phenotypes considered in this study, Figure S4. Minor allele frequency of all missense mutations in: A) HSP90AA1, B) ROCK2, C) IQGAP1, D) CHD4. [file 13073_2019_709_MOESM2_ESM.docx]

| 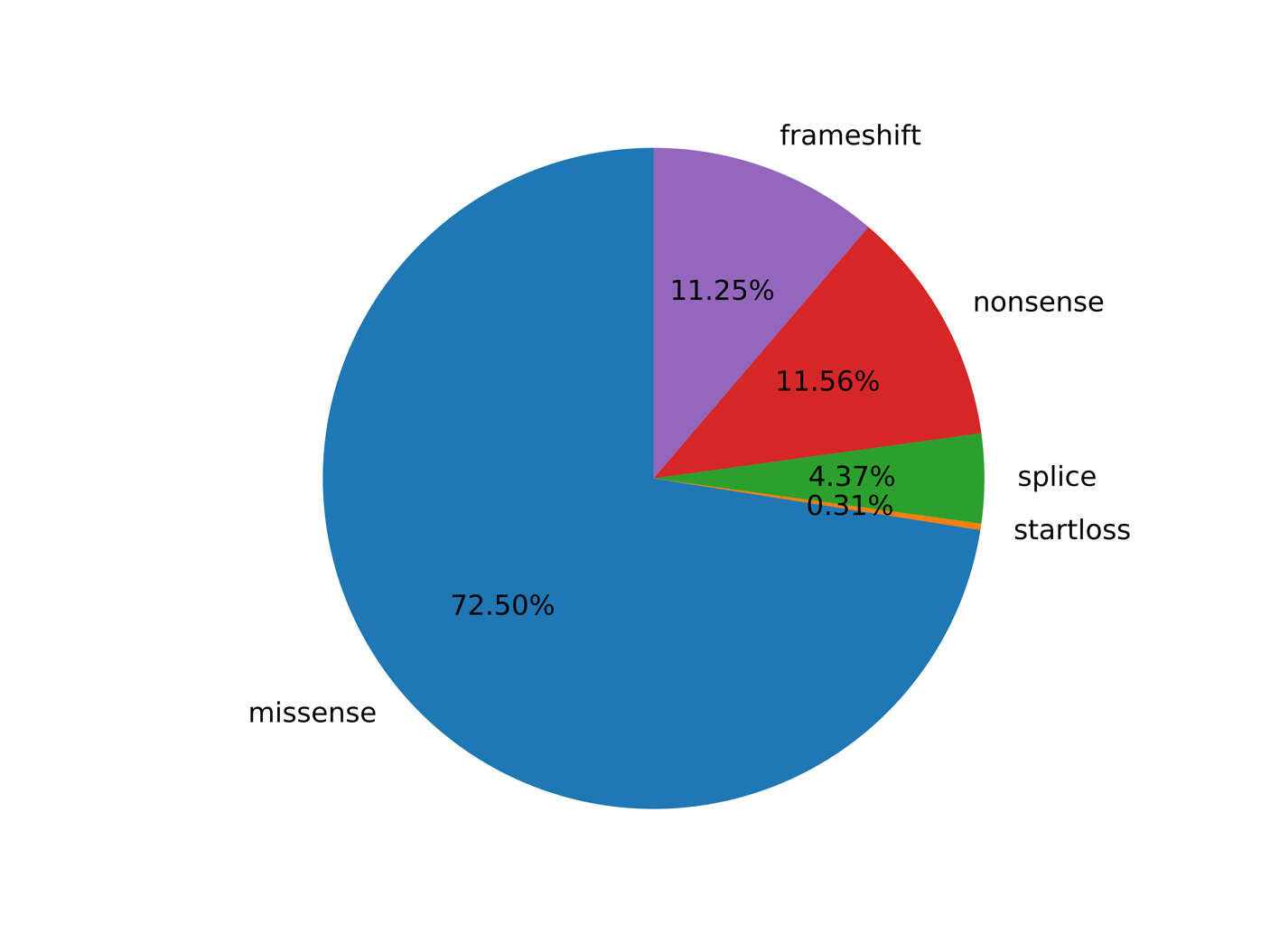 |
| --- |
| Figure S1. Variant types of filtered 320 DNVs in cases |


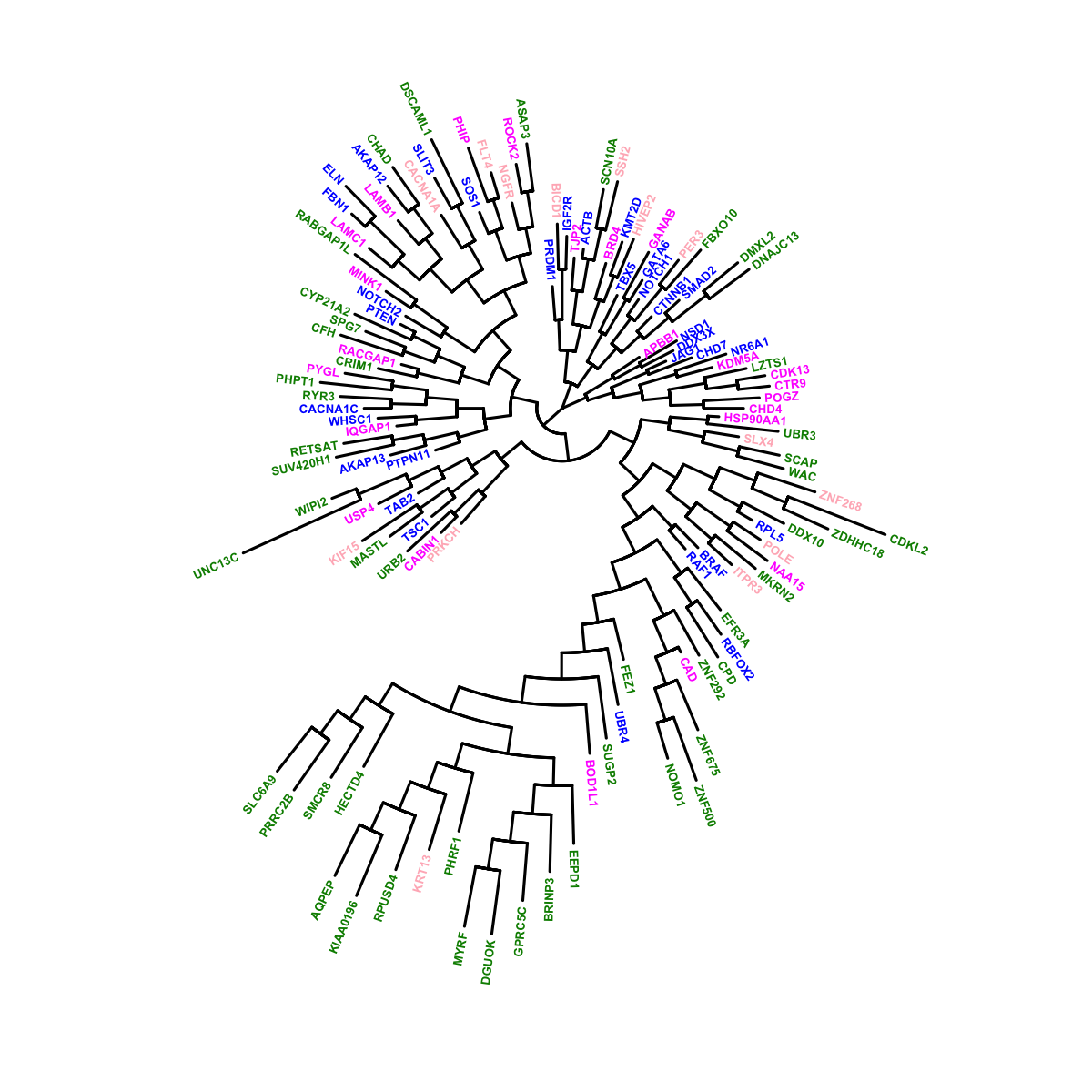


Figure S2. A phylogenetic tree of biological distances between 32 known CHD-causing genes (blue) and 95 candidate genes in cases. The genes with higher priority scores (score>108) are indicated in pink, and the genes with lower scores are indicated in green. Darker shade of pink indicates the genes that are highly expressed in developing heart.

Figure S3. Cardiac phenotypes considered in this study

| 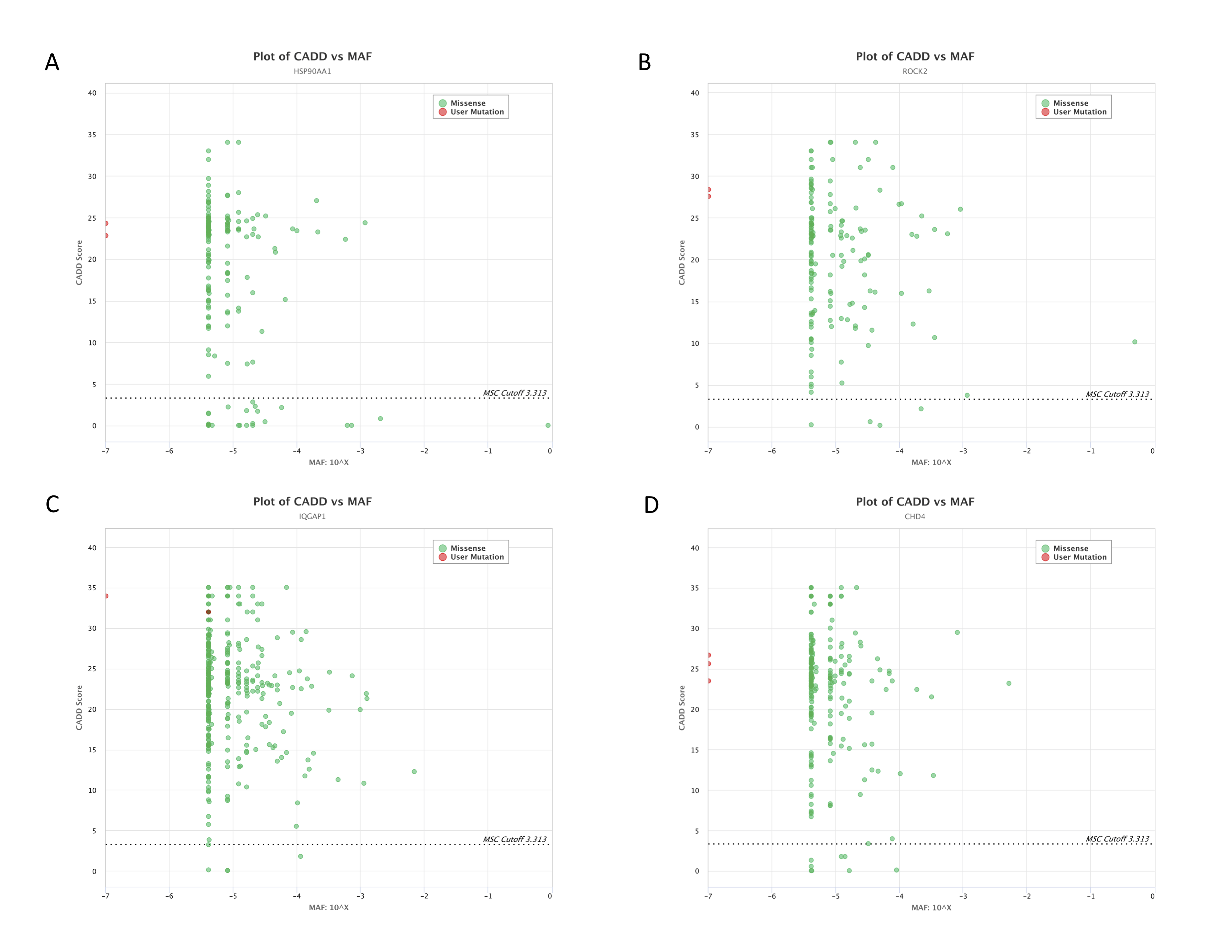 |
| --- |

Figure S4. Minor allele frequency of all missense mutations in A) HSP90AA1, B) ROCK2, C) IQGAP1, D) CHD4 for Non-Finnish European population based on gnomAD database. The dashed line represents the MSC cutoff (95% CI) for each gene, and the candidate mutations are shown in red color.
